# Trichohyalin gene expression is negatively correlated with the severity of dermatitis in a canine atopic dermatitis model

**DOI:** 10.3389/fvets.2024.1396557

**Published:** 2024-08-21

**Authors:** Rosanna Marsella, Kim Ahrens, Rachel Wilkes, Nathalie Munguia

**Affiliations:** Department of Small Animal Clinical Sciences, College of Veterinary Medicine, University of Florida, Gainesville, FL, United States

**Keywords:** dogs, atopic dermatitis, house dust mites, transcriptome, trichohyalin, IL-31 canine atopic dermatitis

## Abstract

**Introduction:**

Canine atopic dermatitis (AD) closely mimics human AD and is recognized as a beneficial animal model. House dust mites (HDM) are a common allergen for both species. The effects of chronic exposure to HDM on the skin have not been studied in this animal model, and it is not known how changes in gene expression correlate to the severity of dermatitis.

**Methods:**

We used an established canine model of AD and took biopsies before HDM exposure (D0) and five times during repeated allergen challenges (on Days 1, 2, 8, 15, and 29, hereafter referred to as D1, D2, D8, D15, and D29). The severity of dermatitis was scored on the same days.

**Results:**

Trichohyalin (*TCHH*) gene expression decreased the most (15-fold decrease on D29 vs. D0) and negatively correlated with the severity of dermatitis. Gap-junction protein gene expression increased over 3-fold on D1, D8, and D29 and positively correlated with the severity of dermatitis. Compared to D0, IL-31 gene expression significantly increased on D8 (*p* = 0.0098), D15 (*p* = 0.0068), and D29 (*p* = 0.0187), but the correlation with the severity of dermatitis did not reach significance.

**Discussion:**

This is the first report on trichohyalin, a protein belonging to the S100 family, and gap-junction protein gene expression in the context of the clinical severity of AD. We propose that these proteins should be further investigated to better understand their role in this complex disease.

## 1 Introduction

Canine atopic dermatitis (AD) shares many similarities with the human counterpart (1), and dogs are a beneficial model for human AD (2–5). The development of canine AD is multifactorial, involving complex interactions between genetics and environmental factors. Exposure to house dust mites (HDM) is a risk factor for AD in both people (6) and dogs (7, 8). Pet dogs have prolonged exposure to indoor allergens (9). Allergic skin diseases are becoming increasingly common in dogs, and factors such as the urban environment and access to upholstered furniture are documented risk factors (10). Similar to humans, the skin of atopic dogs is more permeable (11), and this increased permeability is a risk factor for allergic sensitization (12). In recent years, much emphasis has been placed on filaggrin and its role in AD. While filaggrin mutations do not appear to play a major role in canine AD based on existing studies (13), it is known that inflammation affects filaggrin expression (14). Tight junction proteins have also been considered for their role in skin permeability in canine AD. The chronic exposure of atopic skin to HDM is likely to cause changes in the skin transcriptome that could correlate with dermatitis. Studies requiring repeated biopsies and standardized allergen exposures are best conducted using a model. Published studies on skin transcriptome in dogs after HDM exposure are limited to 24 h (normal dogs) (15) and 48 h (atopic dogs) (16). Both studies biopsied patch test areas where large amounts of HDM were placed under occlusion. Short exposure and occlusion are not representative of real-life exposure, since the exposure typically happens in lower amounts, without occlusion, and for prolonged periods of time. Thus, our study aimed to investigate the skin transcriptome of atopic dogs epicutaneously exposed to HDM over a 4-week course to identify differentially expressed genes that could correlate with the severity of dermatitis.

## 2 Materials and methods

### 2.1 Animals and allergen challenge

**Figure 1:**
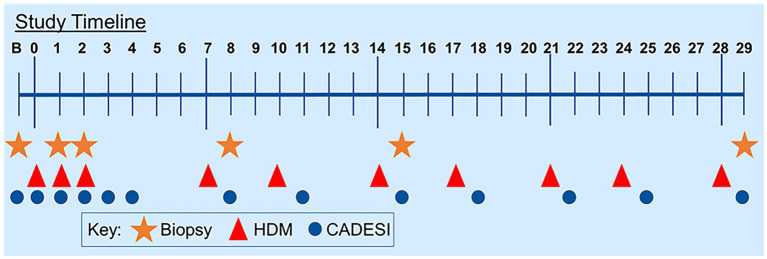
Study timeline. Dogs were challenged with house dust mites for 3 days in a row at the beginning of the study and then twice weekly for the remaining duration of the study.

Five atopic beagles from a colony were used as the model for AD. An allergen solution of a species of HDM (*Dermatophagoides farinae,* Greer Laboratories, 16.5 mg/mL) was applied (1.6 mL/challenge/dog) to the groin and chest daily for the first 3 days and twice weekly for 3 weeks (Figure 1). The dose of HDM was calculated to mimic real-life exposure (dust mite content of a mattress) (17, 18). Protocol was approved by the Institutional Animal Care and Use Committee of the University of Florida (201910621).

### 2.2 Clinical evaluation

The severity of dermatitis was scored using a validated scoring system (Canine Atopic Dermatitis and Extent Severity Index 03 (CADESI-03) (19) on Day 0 (D0), 6 h after exposure on D1, D2, D3, and 24 h afterward (Figure 1).

### 2.3 Skin biopsies

Biopsies (8-mm punch) were taken on D0 (prior to allergen exposure), 24 h after challenge on D1, on D2, on D8, on D15, and on D29 (Figure 1). Each biopsy was taken from lesional areas whenever available. Biopsies were split as follows: Half was frozen in liquid nitrogen for the extraction of genetic material and half was fixed in 3.7% paraformaldehyde.

### 2.4 Histopathology

Paraformaldehyde-fixed sections were stained with hematoxylin and eosin. Slides were scored for the severity of inflammation and the degree of acanthosis (scale 0–3, 0 being normal and 3 being severe). The total score was calculated by adding the scores (16). Scoring was performed by the same investigator who was unaware of the timing of biopsies.

### 2.5 Microarray analysis

Total mRNA was extracted using a 5 PRIME PerfectPure RNA extraction kit (cat no. FP2302500) following the kit protocol (QuantaBio Avantor, Radnor, PA) and quantified using a NanoDrop Spectrophotometer (Thermo Fisher Scientific, Waltham, MA). Complementary DNA was made using SuperScript VILO cDNA Synthesis Master Mix (Invitrogen Life Technologies, Grand Island, NY). The cDNA samples were labeled with biotin and hybridized to the Canine Genome 2.0 Array (GeneChip Hybridization Kit, Affymetrix). Microchips were washed, stained, and scanned using a GeneChip scanner. Reagents were used according to the manufacturer’s instructions (Affymetrix, Santa Clara, CA). Since the chip did not include IL-31, real-time PCR was performed for IL-31 mRNA.

### 2.6 Data analysis

Microarray data were analyzed using Transcriptome Analysis Console Software. Overall, 5% of the genes with the largest fold change were extracted. Differentially expressed transcripts were identified using the *F* test to compare different time points with each other. The false discovery rate test was set at an alpha of 0.05, and FDR-adjusted *p*-values were calculated. Fold changes higher than 2 are reported. PCR data were analyzed using the 2^-ΔΔCt^ method. Correlations between gene expression and dermatitis were calculated using the Pearson correlation coefficient.

## 3 Results

### 3.1 Dermatitis and histology

**Figure 2:**
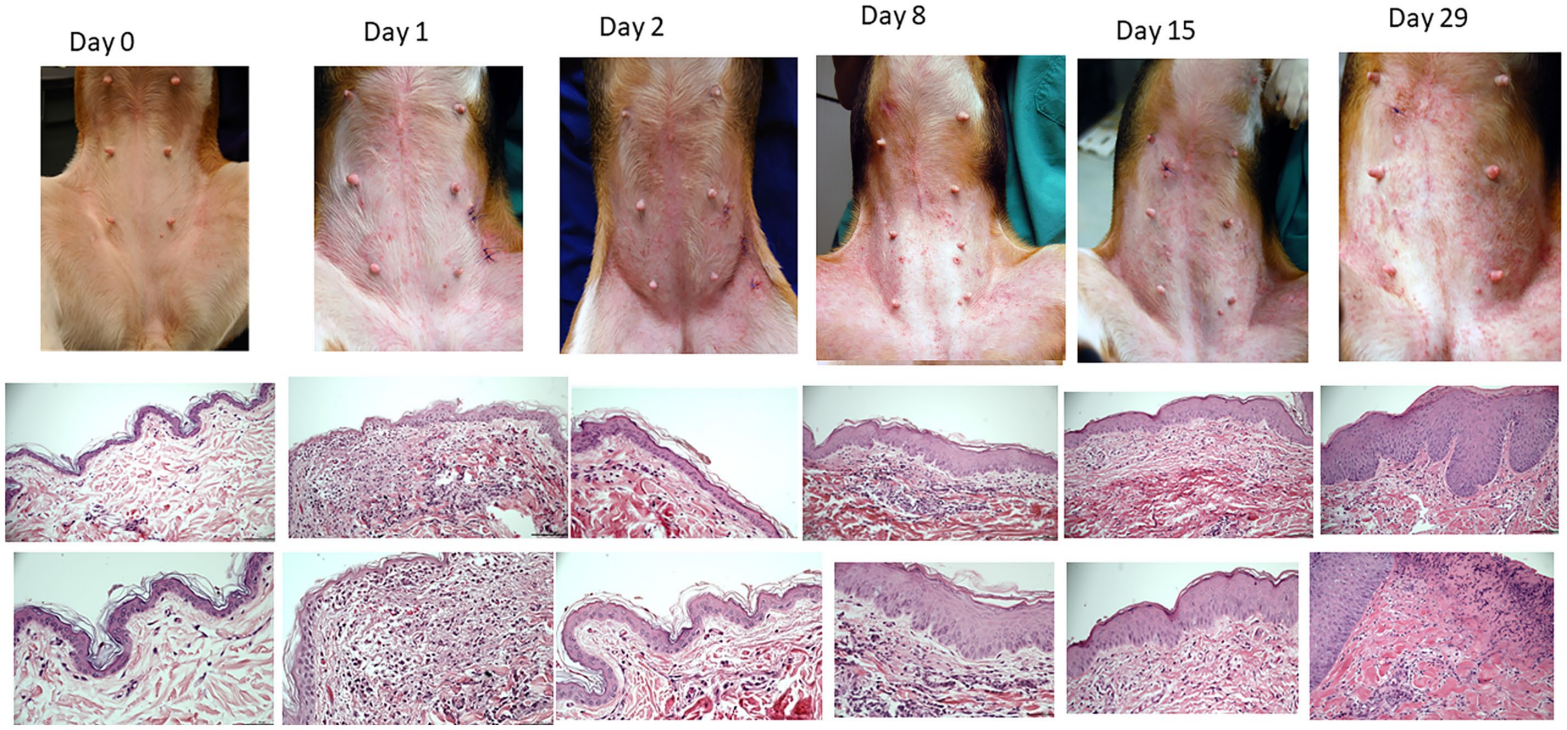
Example of clinical presentations of atopic beagles before the beginning of house dust exposure and throughout the course of allergen challenges (first row). Erythematous macules and papules were the primary clinical lesions. Intense pruritus developed as exposure progressed, leading to secondary excoriations. Representative histopathology of the skin (second row, 20X, third row, 40x). Starting on D1, there was development of a mononuclear and eosinophilic infiltrate in the superficial dermis (second pictures from the left). From D8 onward, progressive thickening of the epidermis was noticeable.

All dogs flared with pruritus and dermatitis after HDM exposure. The severity of dermatitis reached a plateau after 1 week of HDM exposure (Supplementary Figure S1). Examples of clinical lesions and histopathology findings are shown in Figure 2. The most common lesions were erythematous macules and papules. Biopsies showed a progressive increase in superficial perivascular to diffuse mononuclear and eosinophilic dermatitis and epidermal hyperplasia over the course of exposures (Figure 2). Histology scores progressively increased over time (Supplementary Figure S2).

### 3.2 Gene expression changes and correlations with dermatitis scores

Keratin 5 gene expression increased the most (>12-fold increase on D8) and trichohyalin decreased the most (>15-fold decrease on D29). L-amino-acid oxidase-like gene expression increased 5-fold on D1 and D8, 4.5-fold on D15, and 2-fold on D29. Gap-junction protein increased >3-fold on D1, D8, and D29. Calumenin increased 2.5-fold on D1 and D8. These changes were initially calculated as significant, but when FDR-adjusted *p*-values were used, significance was lost. Data on individual dogs are available in Supplementary File S3.

**Figure 3:**
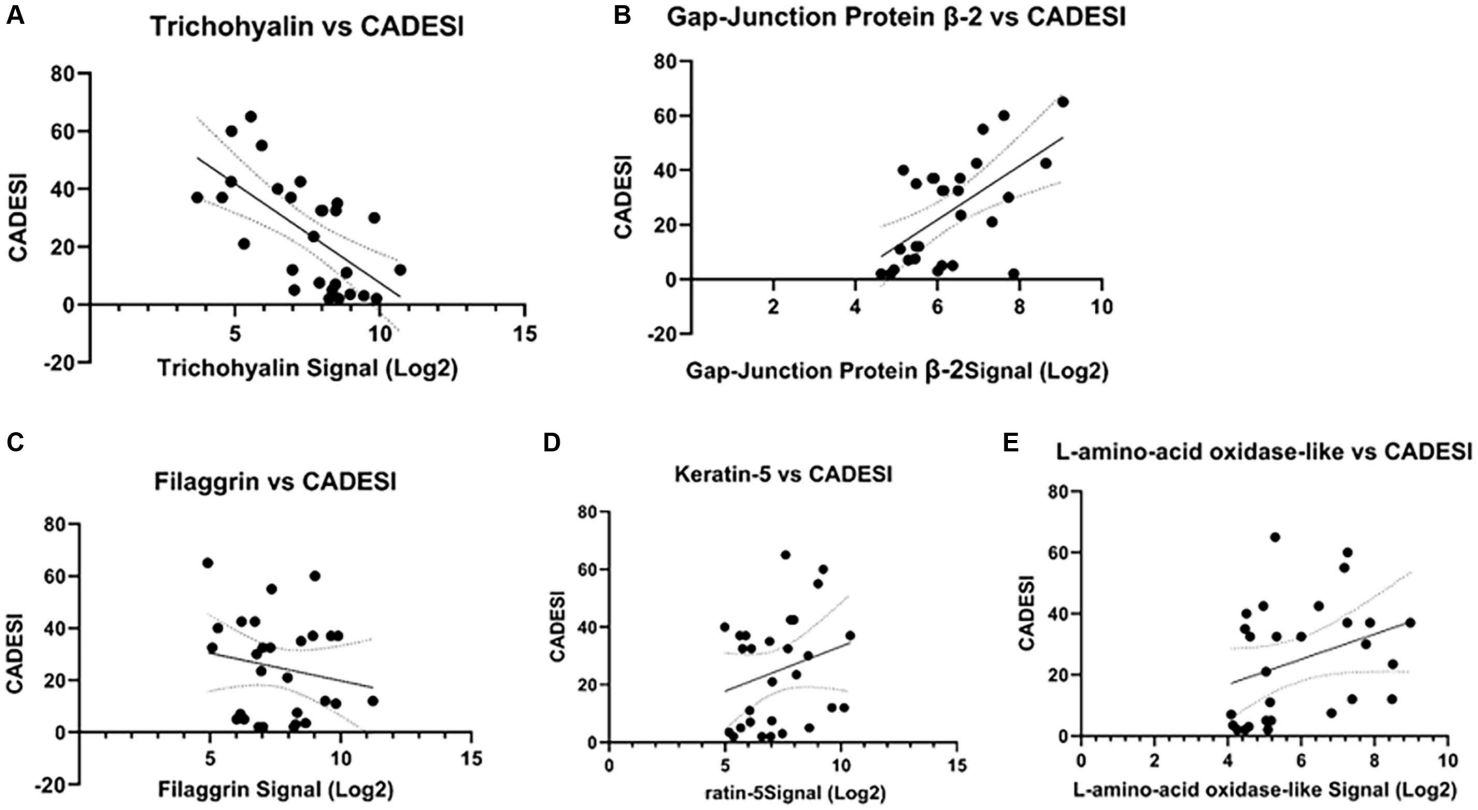
Correlations between selected genes and dermatitis scores (CADESI). **(A)** A statistically significant negative correlation existed between CADESI and trichohyalin gene expression (*p* = 0.00025, *r* = −0.68). **(B)** A statistically significant positive correlation between gap-junction protein and CADESI (*r* = 0.59; *p* = 0.00093) was observed. No other significant correlations were found **(C–E)**.

Compared to D0, IL-31 gene expression increased 4-fold on D1, 3.8-fold on D2, 4-fold on D8 (*p* = 0.0098), >6-fold on D15 (*p* = 0.0068), and > 5-fold on D29 (*p* = 0.0187) (Supplementary Figure S3). The correlation between IL-31 mRNA and dermatitis scores (CADESI) was not statistically significant (Supplementary Figure S4). A statistically significant negative correlation was found between CADESI and trichohyalin gene expression (Figure 3A; *p* = 0.00025, *r* = −0.68). A statistically significant positive correlation was observed between gap-junction protein and CADESI (Figure 3B, *r* = 0.59; *p* = 0.00093). No significant correlations existed between and CADESI and filaggrin, keratin 5, and L-amino-acid-oxidase-like gene expression (Figures 3C–E).

Table 1 presents a summary of genes whose expression changed more than 1.5-fold compared to the baseline. Blue highlights decreases in gene expression, while yellow highlights increases in expression.

**Table 1:** Summary of the genes with a fold change higher than 1.5 when compared to the baseline (Day 0).

Description	D1 vs. D0 fold change	D2 vs. D0 fold change	D8 vs. D0 fold change	D15 vs. D0 fold change	D29 vs. D0 fold change
L-amino-acid oxidase-like	5.13	1.66	5.08	4.36	2.03
Gap-junction protein, beta 2, and 26 kDa	3.26	1.81	3.91	2.23	3.45
Calumenin	2.69	1.99	2.54	1.61	1.47
Uncharacterized LOC102154067	2.1	1.52	1.62	1.52	1.67
Mitochondrial transcription termination factor-4	−1.75	−1.94	−2.27	−1.99	−2.24
Trichohyalin	−1.86	−2.67	−7.35	−2.59	−15.61
Cold-inducible RNA-binding protein	−1.46	−1.25	−1.8	−1.38	−2.18
Keratin 5	6.6	5.54	12.58	1.22	1.45
IL-31	4.1	3.8	4.2	6.5	5.3

Blue highlights decreases in gene expression, while yellow highlights increases in gene expression.

## 4 Discussion

In our small study, the genes whose expression changed the most were involved in the skin barrier. Trichohyalin decreased the most and had a statistically significant negative correlation with the severity of dermatitis. Trichohyalin belongs to the S100-fused protein family, such as filaggrin and cornulin (20). S100 proteins are important for epidermal renewal and differentiation (21). These proteins have been considered in the context of AD (22, 23), with filaggrin being one of the most studied (24, 25). Trichohyalin is important for the mechanical strength of the skin as it is cross-linked to various proteins in the cornified cell envelope of the inner root sheath. It is also present in the stratum granulosum and corneum of the epidermis (26, 27). Although very few studies have focused on trichohyalin and AD, it was reported to play a role in AD in a skin model (28), and variants have been associated with atopic patients (29). This is the first study to report on trichohyalin gene expression in canine AD and to correlate gene expression with the severity of dermatitis.

Gap-junction protein gene expression change was statistically correlated in a positive fashion with the severity of the dermatitis. Gap junctions are protein channels between adjacent cells that allow the passage of ions, potential signaling molecules such as death signals from injured cells, and small metabolites. Thus, gap junctions are important for cell-to-cell communication. Connexins are examples of gap-junction proteins. Connexin plays an important role in wound healing (30), inflammatory responses, and cell differentiation (31). Connexins have been reported to be increased in subacute and chronic eczema in people (32). We have not found any previous reports on the relationship between gap-junction proteins and canine allergic skin disease.

Our study focused on atopic dogs only and did not include HDM challenges in normal dogs; thus, it remains unknown if mites could cause similar changes in normal dog skin. Dust mites are a well-documented cause of atopic sensitization (6), but they have been reported to have no direct pro-inflammatory activity on normal human keratinocytes (33). Previous studies in dogs have shown that the HDM exposure used in this study does not affect the skin barrier in normal dogs (e.g., no changes in transepidermal water loss), while it worsens the skin barrier in atopic dogs (12). Trichohyalin expression is suppressed by Th2 cytokines, similar to filaggrin (28); thus, it is likely that the allergic inflammation stimulated by the allergen challenge (documented by the histopathology findings) played a role.

In our biopsies, we noticed a consistent and remarkable epidermal hyperplasia as the exposure progressed. Keratin 5 gene expression increased by 12-fold after HDM exposure, although these changes did not reach significance due to the small number of dogs used in our study. Keratin 5 is expressed in basal keratinocytes and is important for mechanical anchoring to the desmosomes of basal cells. Keratin 5 plays a role in maintaining cell proliferation in the basal layer and is expressed in mitotically active epithelia (34). The increase in gene expression found in our biopsies reflects the increased proliferative state of the epidermis as a result of the inflammatory response (35, 36). In previous studies, we found that allergic dogs belonging to this colony would react to HDM exposure by significantly increasing the thickness of the epidermis, while normal beagles exposed to HDM using the same protocol would not show the same type of proliferative response (37).

L-amino acid oxidase gene expression changed over 5-fold at various time points, but no significant correlation was found with the severity of dermatitis. L-amino acid oxidase is secreted by antigen presenting cells and Th17 cells (38). Interleukin-4-induced gene 1 (IL4I1) is the best-characterized L-amino acid oxidase that facilitates the differentiation of CD4+ into regulatory T-cells. In our study, we did not observe major fold changes of cytokines except for IL-31. Although there were some fold changes, we did not find a significant correlation between IL-31 and the severity of dermatitis due to the small number of dogs used in our study. Changes in IL-31 gene expression have been reported in acute allergen challenges in other canine AD models (16, 39). IL-31 is a documented important player in both canine (40) and human AD (41, 42) and a target for treatment in both species (43, 44).

One major limitation of our study is the small number of dogs and the fact that normal dogs were not similarly challenged; thus, no conclusive statements can be made on whether the changes observed are specific to atopic skin. Atopic dermatitis is becoming extremely common in pets, and chronic exposure to HDM may be one of the many factors playing a role. It is likely that the damage to the skin done by dust mites in susceptible individuals can lead to a progressive cycle of inflammation and worsening of the skin barrier. It interesting that no significant associations were found between filaggrin gene expression and the severity of dermatitis in our study and that filaggrin was not in the short list of differentially expressed genes in our sequential biopsies. It is possible that our sample size was too small to detect these changes and that a larger number of dogs would have been necessary for that. In the future, dogs of various breeds should be included to identify if filaggrin may play a role in some breeds but not others.

Microarray studies quantifying a large number of genes and making numerous comparisons have a high risk of finding false-positive associations. In our study, while several genes were found to have large fold changes when FDR-adjusted *p*-values were used, these changes did not reach statistical significance. Unfortunately, our colony was the last colony of truly atopic dogs rather than normal dogs artificially sensitized to dust mites and was used as a model of acute inflammation. Our dogs naturally developed AD (e.g., puppies that were adopted out before any laboratory allergen exposure would develop AD as pets once exposed to allergens). The allergen challenges used in the study was a tool to time the development of reactions and correlate the transcriptome changes with clinical dermatitis. The sample size of this study was dictated by the small number of dogs that remained in the colony before the discontinuation of this model.

In summary, in this model of AD, chronic epicutaneous exposure to HDM led to AD flares and epidermal proliferation. A decrease in trichohyalin and an increase in gap-junction protein gene expression correlated with the severity of clinical dermatitis.

## Data Availability

The original contributions presented in the study are included in the article/Supplementary material; further inquiries can be directed to the corresponding author.
