# Supplementary material for: Trichohyalin gene expression is negatively correlated with the severity of dermatitis in a canine atopic dermatitis model
Source: Front Vet Sci. 2024 Aug 21;11:1396557. doi: 10.3389/fvets.2024.1396557 (PMC11371671; doi:10.3389/fvets.2024.1396557)
Supplement: Supplementary file 6 [file Data_Sheet_1.docx]

Supplementary Material

Trichohyalin gene expression is negatively correlated with severity of dermatitis in a canine atopic dermatitis model

Rosanna Marsella,^*^ Kim Ahrens, Rachel Wilkes, Nathalie Munguia

***** Correspondence: Rosanna Marsella: [Marsella@ufl.edu](mailto:Marsella@ufl.edu)

## Supplementary Figures


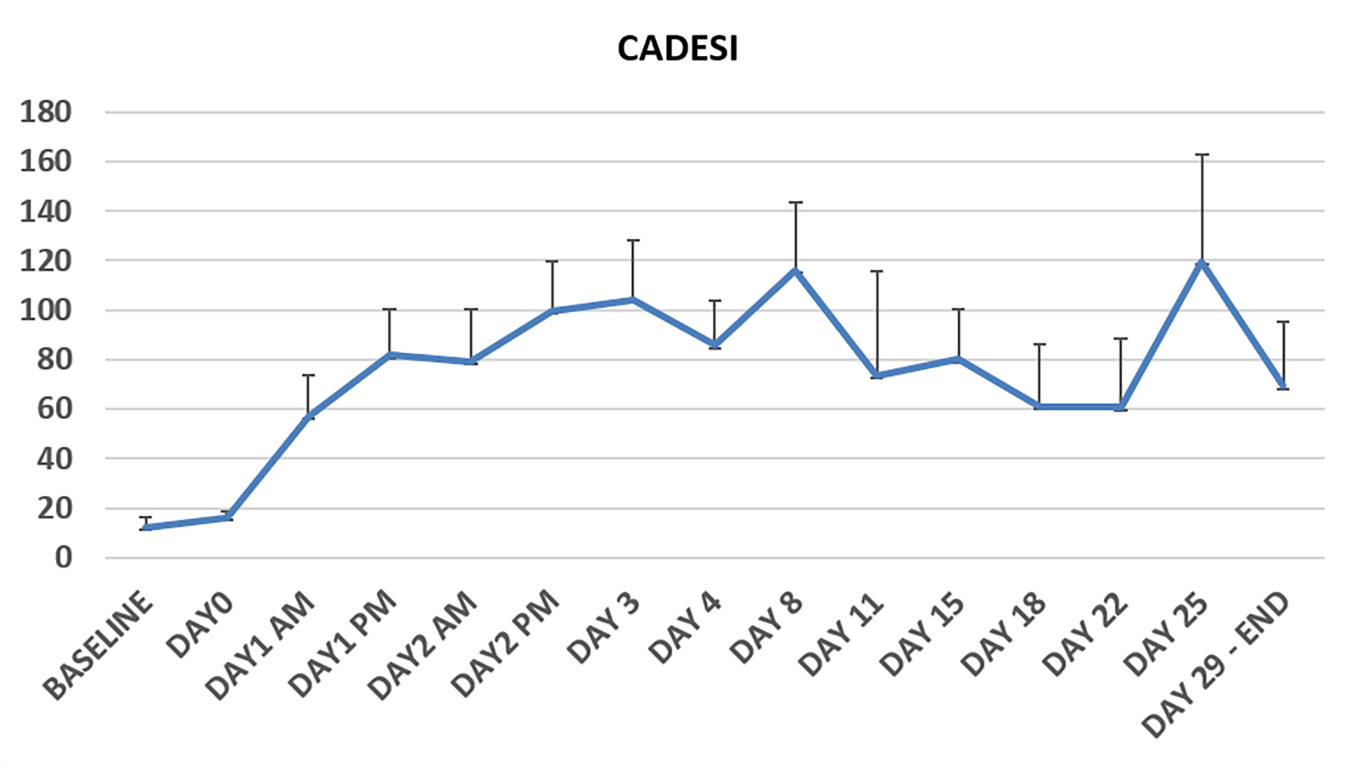


**Supplemental Figure S1.** Means and standard deviations of the CADESI seen in the dogs. Scores show the progressive increase in the first week of challenge and then a plateau, except for the very last week (Day 25) when another peak of severity was noticed.


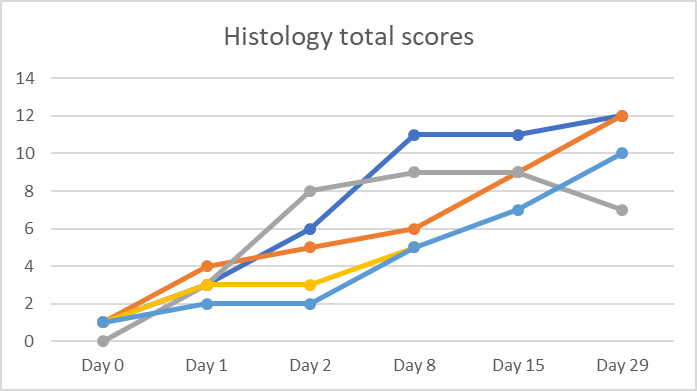


**Supplemental Figure S2**. Total histology scores for each dog as the exposure to the allergen continued over a 4-week period. This score was calculated adding scores for degree of epidermal hyperplasia, dermal edema, epidermal and dermal inflammatory infiltrate.


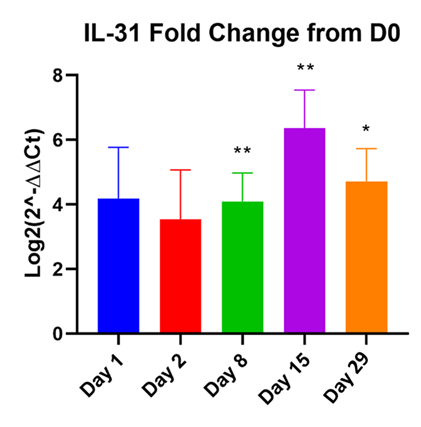


**Supplemental Figure S3**. qPCR data showing mean and standard deviation of the fold change in IL-31 expression from baseline (Day 0). Significant changes were found on D8, 15 and 28 using a one sample t-test. * indicates p < 0.05; ** indicates p < 0.01.


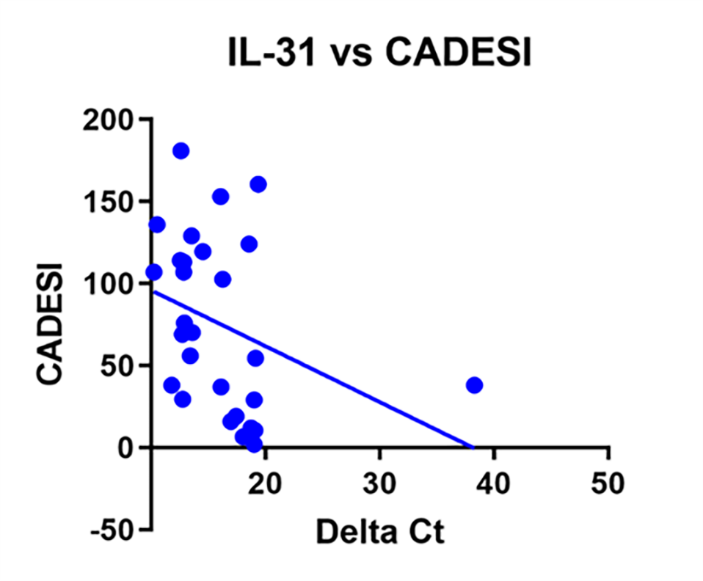


**Supplementary Figure S4**. Correlation between IL-31 gene expression and severity of dermatitis measured by CADESI scoring was not statistically significant.
